# Train your robot in AR: insights and challenges for humans and robots in continual teaching and learning

**DOI:** 10.3389/frobt.2025.1605652

**Published:** 2025-08-13

**Authors:** Anna Belardinelli, Chao Wang, Daniel Tanneberg, Stephan Hasler, Michael Gienger

**Affiliations:** Honda Research Institute Europe, Offenbach, Germany

**Keywords:** long-term human-robot interaction, continual learning, learning from demonstration, teachable robots, augmented reality

## Abstract

Supportive robots that can be deployed in our homes will need to be understandable, operable, and teachable by non-expert users. This calls for an intuitive Human-Robot Interaction approach that is also safe and sustainable in the long term. Still, few studies have looked at interactive task learning in repeated, unscripted interactions within loosely supervised settings. In such cases the robot should incrementally learn from the user and consequentially expand its knowledge and abilities, a feature which presents the challenge of designing robots that interact and learn in real time. Here, we present a robotic system capable of continual learning from interaction, generalizing learned skills, and planning task execution based on the received training. We were interested in how interacting with such a system would impact the user experience and understanding. In an exploratory study, we assessed such dynamics with participants free to teach the robot simple tasks in Augmented Reality without supervision. Participants could access AR glasses spontaneously in a shared space and demonstrate physical skills in a virtual kitchen scene. A holographic robot gave feedback on its understanding and, after the demonstration, could ask questions to generalize the acquired task knowledge. The robot learned the semantic effects of the demonstrated actions and, upon request, could reproduce those on observed or novel objects through generalization. The results show that the users found the system engaging, understandable, and trustworthy, but with larger variance on the last two constructs. Participants who explored the scene more were able to expand the robot’s knowledge more effectively, and those who felt they understood the robot better were also more trusting toward it. No significant variation in the user experience or their teaching behavior was found across two interactions, yet the low return rate and free-form comments hint at critical lessons for interactive learning systems.

## 1 Introduction

As robots become commonplace outside laboratories and factories, there is an increasing need to assess how novice users can intuitively personalize the robot’s skills without any technical knowledge. This is particularly the case for robots supposed to support us in our daily activities, possibly in our private spaces, where they will be required to learn to do something in the way we prefer and with the tools we have at hand. In this scenario, crowdsourcing (Van Waveren et al., 2021; Krishna et al., 2022) would not be an option, and while approaches based on Large Language Models (LLM) are increasingly demonstrating progress (Joublin et al., 2024; Lawley and Maclellan, 2024) they still present pragmatic issues in the context of interactive task learning (ITL) from grounded observations (Laird et al., 2017).

Teaching robots should be easy, intuitive, and engaging. It should provide the user with insights into the robot’s perception, reasoning, and action capabilities so that the user can easily take those into account and adjust their teaching straightforwardly, within a few interactions. In human-human interaction, our inner mental model of a novel acquaintance gets increasingly refined as we interact with them. The same should happen with complex technical systems: even if instructing material can inform the user on how to operate a machine and what to expect, the more the users use the system, the more proficient they become at understanding how it processes information and behaves. Nevertheless, living all of our lives in a social context, that is, in contact with agents with very similar embodiment and cognitive abilities, we rely on many assumptions about what others see and understand about the world. The same cannot, in general, be assumed of robots. Different robots have different embodiments (sensors, actuators) and might rely on different cognitive architectures (scene understanding, manipulation capabilities, etc.), all of which might not be directly apparent on a first encounter (Thellman and Ziemke, 2021; Thellman et al., 2022). Further, to learn from each interaction with the tutor, one-shot, multimodal, incremental learning is required, possibly integrated in a complex architecture dealing concurrently with perception, planning, and motor control (Laird et al., 2017). These issues call for a user-centered approach to feedback during teaching: here we rely on our experience with augmented reality (AR) (Belardinelli et al., 2023; Wang et al., 2023) and on learning from demonstration as a paradigm that does not require any explicit robot programming or instruction from the user (Argall et al., 2009; Billard et al., 2016). In such a framework, we wish learning to happen in an abstract and generalizable way without too much effort and weight on a single demonstration: we developed a symbolic learning approach based on knowledge graphs (Tanneberg and Gienger, 2023) capable of representing and generalizing skills in a semantic, effect-driven way. We present its application in a kitchen world with a relatively simple ontology, where questions by the robot can complement each demonstration and expand gained knowledge to similar objects. Finally, the user should be able to assess what the robot has learned and possibly correct or integrate what the robot does right after the demonstration. To this end, in our framework, users can ask the robot to provide a plan to achieve a certain goal, which the robot demonstrates via a symbolic task planner relying on its generalized knowledge (Hasler et al., 2024). Such an end-to-end learning and acting system, tailored to our robotic platform but also seamlessly integrated with AR glasses, allowed us to test the whole interaction flow with naive users in an organic way, without resorting to a Wizard-of-Oz experimental design. Several relevant questions indeed have arisen in recent years when considering robots learning in interaction and how humans approach teaching robots (Thomaz and Breazeal, 2008; Vollmer and Schillingmann, 2018; Sigaud et al., 2022), regarding what makes a good teacher and which communication channels are appropriate. Yet it is not clear what constitutes an engaging and effective teaching paradigm for continual learning, how users go about it when they have to train a robot, and whether they can develop strategies for it depending on their engagement and understanding. In our case, we were interested in investigating how users would perceive and understand the system when interacting “in the wild”, in an unsupervised manner and in real-time. Since both the user’s mental model of the robot and the robot’s capabilities change across multiple interactions, we were interested in characterizing such interdependent temporal evolution. To this end, we conducted an exploratory study to gain insights into such questions. Our contributions here include the presentation of the technical system, the setup and procedure for the user study, and the analysis of the results, along with a discussion concerning insights and limitations. While the design of such a system is specific to our setup and relies on particular design and algorithmic choices regarding communication in Human-Robot Interaction, skill learning, and semantic planning, we believe that its testing in an unconstrained setup–which lets users find out their own teaching strategy–can significantly advance the discussion on critical aspects of human-robot interaction with continuously learning systems (Irfan et al., 2021).

## 2 Related work

In recent years, as all capacities for cognitive robotics have improved, from perception and action to learning and reasoning, research on teachable robots in interactive task learning has increased, along with discussions on the quality of human teaching and robot feedback and how to optimize the teaching interaction (Cakmak and Thomaz, 2014; Vollmer et al., 2014; Vollmer et al., 2016; Ramaraj et al., 2021). As said, imitation learning offers a straightforward way to acquire physical skills. At the same time, other approaches have seen human tutors provide feedback in reinforcement learning frameworks, hence determining the reward for the learner (Thomaz and Breazeal, 2008; Chisari et al., 2022). This also puts quite a burden on the tutor to provide exemplary demonstrations, capture the learner’s feedback, or provide accurate feedback, often without effective means to investigate the robot’s internal representations and understanding. To extend and generalize this type of tutoring and make the best use of the teacher’s common sense and knowledge, the fields of Active Learning in machine learning (Amershi et al., 2014) and Interactive Task Learning in robotics (Mohseni-Kabir et al., 2015; Chao et al., 2010) have offered solutions for the system to proactively ask for human input on some critical cases. Mohseni-Kabir et al. (2015), for example, focused on a single (hierarchical) task, learned from a single demonstration, while the robot asks questions and makes suggestions to figure out the task structure. Chao et al. (2010) had the robot signaling uncertainty about the presented shape configuration in order to request more informative examples. The non-verbal cues yet were not always correctly interpreted, while results suggested active learning controlled by the robot should also be more balanced with the teacher’s wish to lead the teaching. Fitzgerald et al. (2022) proposed an approach for the robot to choose flexibly the best way to query a teacher (i.e., requesting a demonstration, asking for preference, correction, or binary reward) but the benefits were demonstrated in trajectory learning with an oracle teacher, rather than with non-expert humans in real tasks. Furthermore, such approaches, while providing substantial evidence for the effectiveness of interactive learning, both for the robot and the user, do not consider the long-term perspective, where tutors might be interested in incrementally teaching robots across repeated interactions, which is critical when considering personal robots in home environments.

Typically, long-term studies have been conducted in the field of social robotics (Leite et al., 2013), mostly considering acceptance, adaptation, and personalization aspects (De Graaf et al., 2016; Ahmad et al., 2017; Irfan et al., 2020). Often, non-physical tasks are considered, not involving continual learning or focusing on the robot’s social cues and appearance (Perugia et al., 2021). Continual or life-long learning has recently come to the foreground in research, mostly to counter catastrophic forgetting in machine learning (Li and Hoiem, 2017; Parisi et al., 2019), but there is less evidence on how such algorithms transfer to robotic agents in the real world (Lesort et al., 2020). In recent cases where continual learning is applied end-to-end in repeated interactions, it often focuses on learning motion trajectories from kinesthetic demonstrations (Auddy et al., 2023) or learning new objects (Ayub et al., 2024). Nevertheless, considering more advanced scenarios and non-expert users teaching household tasks, a more generalizable, skill-driven, semantic learning would be required. To some extent, semantic level approaches were used for online learning (e.g. (Suárez-Hernández et al., 2021)) and, more recently, for learning from demonstration high-level goals (Sen et al., 2024) but were not tested in an actual human-robot interaction study. Crucially, in Ayub et al. (2024), the authors moved from what is often a more robot-centered approach to a more human-centered one, focused on investigating how users perceive continuously learning robots and possibly adapt their teaching strategies across interactions.

In light of the above considerations, in this study, we had a double goal.

1. On the system side we consider as specific research questions three of the challenges in ITL pointed out by Chai et al. (2019), i.e.,:

• RQ1: How can a robot effectively acquire robust models from a limited number of demonstrations and minimal human guidance during interactive learning?

• RQ2: How can a robot leverage prior experience to adapt and transfer learned models to novel cases, possibly with minimal human effort?

• RQ3: How can an agent make its internal representations—such as causal relationships—understandable and interpretable to human users?

Thus, our first aim was to design a system that could learn new, structured tasks interactively, both from demonstrations and active questions. The focus here is on explainability by intuitively communicating and demonstrating perceptual capabilities and learned semantic skills (Habibian et al., 2023).

2. Adopting a user-centred approach, we were interested in testing early on and in a simplified, yet naturalistic and unconstrained setting, how the functional and interactive capabilities would affect the user experience (UX) and acceptance of the system through an exploratory study. We considered that important factors for UX in the interaction with such a system would be how engaging the whole teaching process is, how understandable would be for the user the way the system processes the collected data and learns, and how trustworthy the learning system appears to be (Alenljung et al., 2017; Moorman et al., 2023). Trust in these cases is dynamically updated as people assess the learner’s progress (Chi and Malle, 2023). Thus, we had the following hypotheses:

• HP1: The more interactions, the lower the engagement (novelty wears off, as shown in the literature (Leite et al., 2013; De Graaf et al., 2017)).

• HP2: The more interactions with the system, the higher the perceived understanding and trust.

We explain next the system architecture and the experimental design.

## 3 System design

Our system relies on the integration of multiple modules for semantic learning from demonstration, skill generalization, symbolic planning, and motion control. These communicate via ROS with the AR framework, which handles the rendering of the scene and of the robot, the interaction with the user, and the sensory input for the learning^1^.

### 3.1 AR functionalities

**FIGURE 1:**
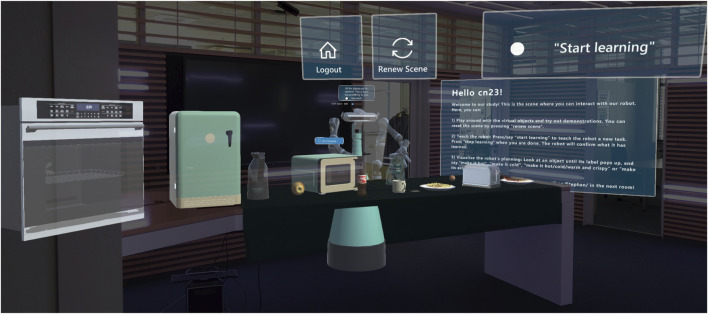
The AR scenario for the robot training and interaction with the user. The scene is a common office space with a table upon which holographic kitchen items are displayed. Virtual controls (to log out, reinitialize the scene, and initiate a teaching episode) were displayed on the upper visual field of the person wearing AR glasses and moving rigidly with the user’s head.

**FIGURE 2:**
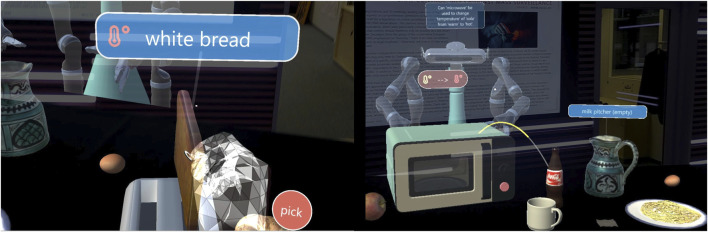
Left: Design elements for online feedback to the tutor about recognized actions and objects. Right: after the demonstration, the robot asks questions to generalize the demonstrated skill (here, after seeing heating milk in the microwave, it asks the tutor *“Can microwave be used to change the temperature of cola from warm to hot?*“) along with virtual graphic overlays.

The shared environment (Figure 1), accessible through AR glasses, includes a kitchen table with multiple appliances and food items, while the robot is displayed on the other side as observing the scene and the user. Object poses are continuously tracked and sent to the back-end system, along with the user’s head pose and performed actions (picking/dropping objects, turning on/off appliances, pouring beverages). Through gesture recognition and grasping/manipulation logic, users interact with virtual objects similarly to how they do with real ones. For example, by detecting collisions between the tracked hand and virtual handles or objects, along with pinching hand gestures, the system recognizes opening or grasping actions and the corresponding object moves along with the hand. To increase explainability and give the tutor online feedback about the robot’s situation understanding, several virtual design elements (Walker et al., 2023) are displayed in AR: object and action labels (XAI cues) are popping up whenever the user gazes at some object or a manual action is recognized, while action labels are also spoken out verbally (see Figure 2, left, see also (Wang and Belardinelli, 2022; Wang et al., 2023; Belardinelli et al., 2023)).

### 3.2 Learning

**FIGURE 3:**
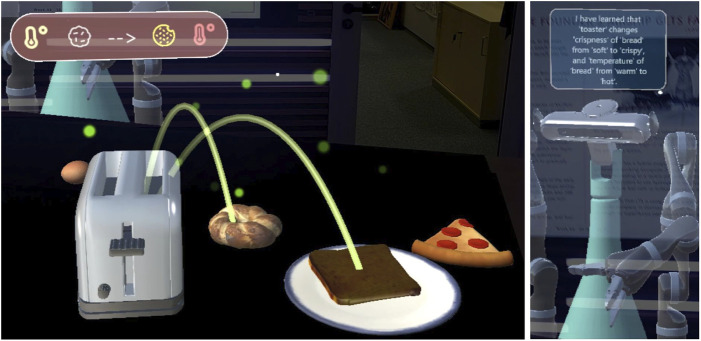
Graphical elements used to explain to the user that the robot learned that food items in the “bread” category can be heated up in the toaster (left). The same message is also provided by text (right) and speech.

For the robot to learn new skills, a two-stage learning concept is used. First, the user demonstrates a skill to the robot, which acquires it using semantic skill learning concepts. The symbolic representation of the skill encodes preconditions, actions, and effects (see Tanneberg and Gienger, 2023, for more details). In this study, there are three types of effects the robot can observe as a consequence of the user’s actions on given appliances and food items: the change of temperature, the change of crispiness, and the change of aroma^2^. This is communicated back to the teacher by uttering a sentence in the form ”I’ve learned that <tool> can change the <attribute> of <food item> from <predicate 1> to <predicate 2>”, with attribute∈{temperature,crispness,aroma} , while tool and fooditem are one of those listed in Table 1, top. As to the predicates of the attributes, temperature can be one of {cold,warm,hot} , crispiness in {soft,crispy} , while aroma can take one value in {noaroma,mint} . In the second stage, the robot asks the user questions about the demonstrated skill to generalize to similar objects. For example, after seeing a demonstration on how to use the microwave to heat milk, the robot can ask whether a similar food (see Figure 2, right) can be heated in the microwave. Across demonstrations and questions, the system generalizes to categories higher up in the WordNet hierarchy (Fellbaum, 2010) (e.g., learning that the toaster can make “bread” hot, which is the category encompassing white bread and bagel, see Figure 3).

**TABLE 1:** Ontological description of the kitchen world used in this study (top), with all semantic skills that could be learned in interaction (bottom). In total, our system handled 25 physical entities, seven types of attributes (elementary and spatial) and eight manipulation object-related actions in a multi-level representation.

Role	Physical entities
Tools	human, hand, robot, oven, refrigerator, microwave, toaster, teabag, milk pitcher, water pitcher, mug, bottle, plate, small plate
Food items	white bread, bagel, apple, spaghetti, egg, pizza, cola, milk, water

Learnable skills			
Tool	Temperature	Crispness	Aroma
Microwave	apple, milk, spaghetti, cola, water, white bread, bagel, egg, pizza		
Oven	white bread, bagel, apple, spaghetti, egg, pizza	white bread, bagel, pizza	
Refrigerator	apple, milk, spaghetti, cola, water, egg, bagel, white bread, pizza		
Toaster	white bread, bagel, apple, spaghetti, egg, pizza, cola, water, milk	white bread, bagel, pizza	
Tea bag			milk, water, cola

### 3.3 Planning

The use of elementary, domain-agnostic attributes helps generalizing skills, allowing the robot to plan to reproduce the observed tasks with the same or similar food items. Beside the elementary attributes introduced above, the system makes use of spatial attributes for content∈ *{inside, outside}* and distance∈ *{close, distant}*, and of device-related attributes for openness∈ *{open, close}* and activity∈ *{on, off}*. In this way, the system can also represent the effects of manipulation actions modifying spatial and device attributes, such as *put in, get out, approach with, pour, open, close, switch on, switch off*. The actions that change spatial predicates are mostly independent of elementary predicates, and the actions that change device-specific predicates are fully independent of spatial and elementary ones. Such multi-level representation relying on different attribute spaces allows a symbolic planner to iteratively plan according to different views, each containing ever more composite sets of attributes, first considering just which objects are present and the predicates of the elementary attributes, up to including spatial relations and device-related predicates (see (Hasler et al., 2024) for more details).

## 4 Study design

The study was designed to be conducted semi-in-the-wild in an office setting, with participants taking part spontaneously, without experimenter supervision (if they wished so). This was meant to allow users to interact with the system as long and as often as they wanted, reinforcing the perception of a 24/7 functioning system. The study was configured as a loose repeated interaction study, with users asked to engage in multiple teaching and planning sessions so that we could monitor the evolution of their behavior and of their subjective experience as the system increasingly learns.

### 4.1 Procedure and methods

**FIGURE 4:**
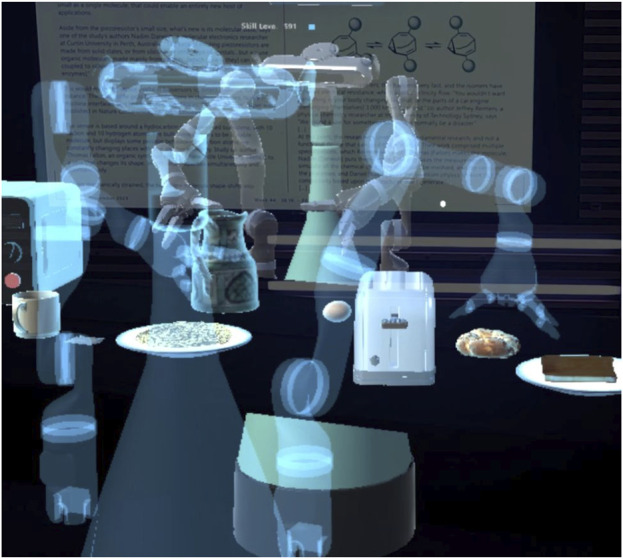
The robot avatar executing the plan it has generated upon request from the tutor.

The study took place in a lobby connecting different office spaces and featuring a long table upon which the virtual scene was calibrated. Participants were invited to the study via mail, explaining the purpose of the study and providing a link to a video tutorial of the experimental procedure so they knew what to expect. Further, flashcards explaining all the steps for the interaction and tips for successful grasping in the AR were displayed on a wall in the lobby. Participants were also reminded by a virtual board in the AR of the basic functions of the system and that they could ask the experimenters in a nearby room in case they needed any assistance (Figure 1 on the right). The informed consent form for the study was provided on the table, along with another form asking for demographic data (gender and age) and the Affinity for Technology Interaction (ATI) scale (Franke et al., 2019). These paper forms were inserted in sealed boxes, before the very first interaction. On the table, participants could find the AR glasses (MS HoloLens) and a laptop to fill out the scale questionnaires after each session. Their answers to the paper and online questionnaires and their interactions with the system were tracked via a pseudo-ID. After logging into the HoloLens, users were presented with the virtual scene in Figure 1 and could play around with objects to get acquainted with virtual object manipulation. The robot would ask the user to teach it something or give it a task to execute. Each robot utterance was also displayed as a speech bubble on top of the robot’s head until a new utterance was spoken out, helping participants remember what the robot said last. Participants were told that their task was to train a personal robot, by demonstrating the use of kitchen appliances to prepare some food, answering related questions, and, to assess the learning progress by asking the robot to make something “hot/cold/crispy” or to change the aroma to “mint”. A teaching episode was started by pressing the “Start learning” button in the user’s upper field of view. Just one skill (e.g., toast bread in the toaster) or multiple skills (e.g., heat water in the microwave and then make tea by putting the teabag in the cup) could be demonstrated. As soon as the user pressed the “Stop learning” button, the robot would give a summary of the (last) learned skill (see Figure 3). Subsequently, a loop would start with the robot asking permission to ask a question and, upon affirmative answer, asking the user a yes/no question (see Figure 2, right). A planning episode could be triggered by the user looking at a food item and uttering their request (e.g., *“Make it hot”*, while fixating the pizza slice). If the robot could find a plan to achieve the desired effect, it would state “This is my plan”. Then, a transparent avatar of the robot would appear on the user’s side of the table (Figure 4) and demonstrate the plan while announcing each step. The plan could reflect the user’s demonstration or deviate from it, in case the robot had generalized (e.g., it could use the microwave instead of the toaster). If no plan was found, this was communicated to the user. The system behavior is handled by a state machine with three states: a default playground state to play with the scene, respawn it to its original state, or log out; a teaching state triggered by the learning button press; a planning state, triggered by a plan request.

To nudge the user to teach sensible skills and answer questions diligently, we introduced a teaching score, which was shown as a number and a progress bar above the robot in the playground state. When logging in for the first time, a zero score is displayed, which is then updated after each teaching episode. Participants were informed that demonstrations and answered questions would increase the robot knowledge as displayed by the score. They were encouraged to increase the score, but they were not aware of the upper bound (i.e., when training was complete). The score is computed as an $F_{\beta }$ score on a predefined ground truth, based on the ontology presented in Tab. 1, bottom, and related generalizations according to the WordNet categories:

$$F_{\beta }=\frac{\left(1+\beta^{2}\right)TP}{\left(1+\beta^{2}\right)TP+\beta^{2}FN+FP}$$

Here, true positives (TP) are correct generalizations–covering the correct objects with the generalization –, false positives are wrong generalizations (FP) – covering incorrect objects –, and false negatives are missing generalizations–not covering objects that should be covered. We set β=2 , thus weighing recall higher than precision. Apart from giving the experimenter an objective measure of the teaching effectiveness, the score represents a gamification element that gave participants some measure of how their teaching expanded the robot’s knowledge and enticed them to keep training the system, also in competition with other users (Sailer et al., 2017; Robinson et al., 2019). It was specified in fact that the pseudo-IDs of the best robot trainers would be ranked and made known at the end of the study.

After logging out of the system, participants were finally prompted to fill out the online questionnaires on the laptop on the side of the table.

Participants were told they could interact as many times as they wanted as long as the experiment was running. They were just recommended not to stay longer than 20 min at any time, if other people were waiting to interact, thus they were deciding themselves how long and how often to participate. Prototypical interactions are exemplified in the videos in the Supplementary Material.

### 4.2 Measures and analyses

We used established and validated scales, aimed at assessing the user experience through the proxy constructs of engagement, understanding, and perceived trust: the User Engagement Scale (UES) (O’Brien et al., 2018), in the short format and with the three subscales of interest for us, i.e., Focused Attention (UES-FA, three items), Perceived Usability (UES-PU, three items), and Reward (UES-RW, 3 items), all evaluated on a five points Likert-scale; the Subjective Information Processing Awareness Scale (SIPAS, six items, six points Likert-scale, Cronbach’s α=0.91 ) (Schrills et al., 2021); and the Trust Perception Scale - HRI (THRI, 14 items, 0%–100% rating scale, in 20% intervals) (Schaefer, 2016). We reasoned indeed that in the context of long-term HRI such constructs would play a large role in the decision of the user to keep engaging with the robot, besides the actual utility and effectiveness of the robot. The user study went on for about 13 weeks (with season holiday interruption). The whole framework was active and available during working hours 5 days/week. In the end, the data collected amounted to the subjective measures (the questionnaires) and behavioral data collected through the system and stored in a database. This latter data comprises for each user and single interaction: the trained model, the score achieved, timestamped teaching episodes in terms of learned skills, questions and answers, and timestamped planning episodes in terms of user requests and related plan or negative answer. Since this was an exploratory study, we looked here at descriptive data and correlations between subjective measures, behavioral data, and teaching performance.

### 4.3 Participants

Participants were recruited among associates and PhD students at our institute. In total, 37 people participated in the study, three of which did not fill out the demographics and ATI questionnaires and one did not fill out the post-interaction questionnaires. Thus, we analyzed the data from 33 participants (26 male, seven female; age between 26 and 59, M = 36.03). The median ATI score was 4.55 (SD = 0.82). Thus, participants were non-experts as to AR or the specific functioning of the system, but were tech-savvy and often with an engineering background.

## 5 Results

Overall, 33 people interacted with the system at least once; of these, 16 interacted twice, and only two people used the system in three or more sessions. We first look at what people did with the system across all interactions by some descriptive measures and then at how participants subjectively perceived the system. While each single interaction could consist of multiple teaching, questioning, and planning episodes, subjective data were collected only once per interaction. For this reason, we look separately at subjective measures after the first and the second interaction and how considered measures changed for those who used the system again. For descriptive metrics of Likert scales, we report median, mode, and range and test them using non-parametric tests, even when normally distributed, as recommended in (Schrum et al., 2023). These latter analyses were Bonferroni corrected. We further looked into the correlation between collected variables to gain insights into possible relationships.

### 5.1 System interaction analysis

As to behavioral measures that might be insightful about how participants interacted with the system, we computed for each participant and each interaction the following measures: the number of demonstrated skills, the number of answered questions, the final score, and the number of requested plans.

**FIGURE 5:**
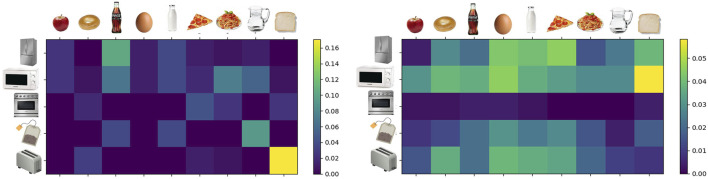
Normalized contingency tables for the demonstrated skills (left) and the answered questions (right).

**FIGURE 6:**
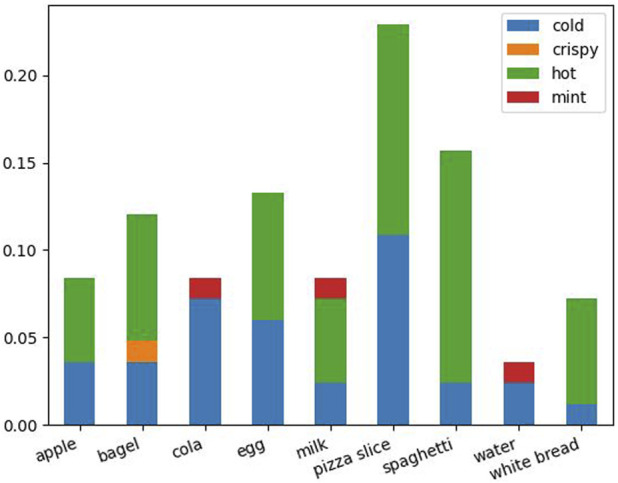
Stacked bar graph of all planning requests across users (normalized). On the x-axis all available food items are listed. The different stacks represent the proportion of each requested effect (e.g., for the apple, users requested the robot to make it either hot or cold).

We collected such data across all participants and interactions to get an overview of what skills were mostly taught and which plans were requested more often. In general, each participant taught at least one skill and answered some related questions. Figure 5 shows the normalized distribution of the overall 164 skills that were demonstrated and 463 questions that were answered as pairs <tool,fooditem> . It can be seen on the left that toasting the bread with the toaster was by large the most demonstrated skill, probably because it was the one provided as an example in the video tutorial. Since the toaster, the microwave, and the fridge were the devices demonstrated more frequently, these were also the ones occurring most frequently in the questions (Figure 5, right). Considering the requests to prepare some of the food items, only 20 participants made use of this feature. Overall, 84 requests were made to the robot, distributed as depicted in Figure 6. Still, while successful plans were displayed by the robot avatar in 23.4% of cases, in most cases the robot was not able to execute a full plan for a number of reasons: no plan was found because no generalization was available (56.4%) or the effect was already achieved (i.e., the scene was not reset after a demonstration, 8.5%), while in 11.7% of cases a plan was found but not executed to the end. The reason for this latter case was that while the high-level (semantic) generalization was correct, which was the focus here, the low-level manipulation would not transfer to a new device. For 19 (23%) plan requests, the user had not previously demonstrated the same skill nor answered a specific question involving the same effect and the same food. Hence, presumably, they assumed the system had already generalized their demonstrations or answers to entire food categories (which was actually the case only for six of such requests for which a plan was devised).

### 5.2 First interaction results

**FIGURE 7:**
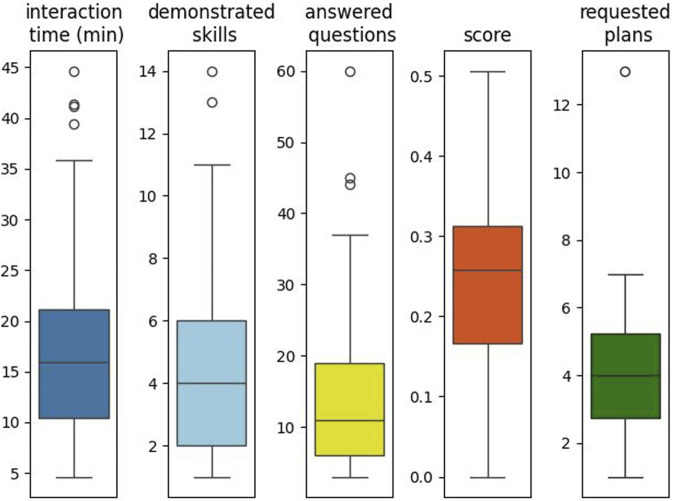
Boxplots of overall interaction measures with the system, across all interaction sessions. The Requested Plans subplot here considers only those users who requested at least one plan from the robot (N = 20).

To further characterize how single participants interacted with the system, we looked at descriptive statistics of the most relevant behavioral indicators, shown in Figure 7. Participants spent overall an average of 18.34 min (SD = 10.56) interacting with the system, demonstrated 4.7 skills each (SD = 3.3), answered 15.8 questions (SD = 13.6), and reached an average score of 0.25 (SD = 0.12). It must be recalled at this point that the F2 score considers all possible effect generalizations (e.g., not just that the oven can make warm food hot but that it can also make cold food warm). In most cases, demonstrated skills involved warm food (the neutral level of the temperature attribute) made either cold or hot by the related device, but rarely users put a hot food item in the refrigerator to make it warm. For this reason, the highest reached score was 0.5, and the average score was relatively low. Considering just the people who requested the robot to devise at least one plan, on average, 4.7 plans were requested (SD = 3.1).

#### 5.2.1 Subjective measures

**FIGURE 8:**
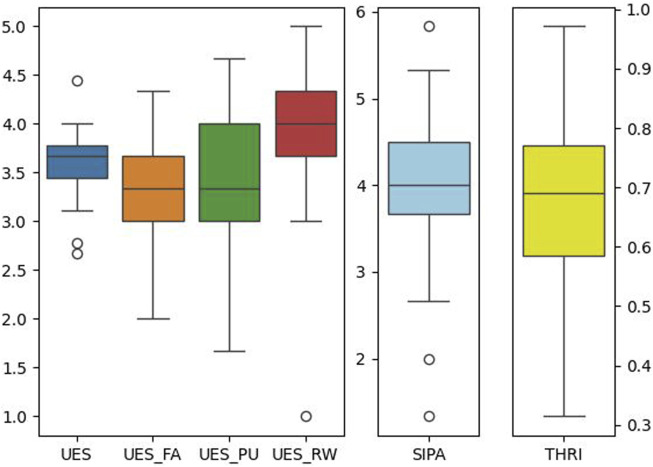
Boxplots of questionnaire results after the first interaction (N = 33). Left to right: User Engagement Scale with its three subscales (Focused Attention, Perceived Usability, and Reward) evaluated on a five-point scale; Subjective Information Processing Awareness Scale (6-points); Trust Perception Scale-HRI (percentage scale).

When considering only the first interaction, the descriptive statistics regarding the UES, SIPAS, and THRI scales are presented in Tab. 2, along with the normality analysis. A one-tailed Wilcoxon signed-rank test confirmed that the median value was significantly above the “neutral” midpoint for UES (coded as 3.0) and all its subscales and the border between “slightly disagree” and “slightly agree” for SIPAS (coded as 3.5). Similarly, the percent of time users would generally trust the robot was higher than 50% for the THRI scale (cfr. Table 2; Figure 8). No significant correlation was found with the ATI scores (all p>.05 ).

**TABLE 2:** Descriptive statistics for the three used scales (after the first interaction). For each scale the number of Likert-scale points or rating range is reported in parentheses. All scales and subscale means were significantly above the neutral point (cf. last column right).

	Descriptive			Normality		>Midpoint	
	Median	Range	Mode	W	p	W	p
UES (5 points)	3.67	1.78	3.67	0.964	0.340	550.0	<.001
Focused Attention (UES-FA)	3.33	2.33	3.33	0.926	0.027	322.5	<.001
Perceived Usability (UES-PU)	3.33	3.00	3.00	0.957	0.214	318.0	0.006
Reward (UES-RW)	4.00	4.00	4.00	0.774	<.001	497.0	<.001
SIPAS (6 points)	4.00	4.50	4.00	0.968	0.419	416.5	0.012
THRI (0–1)	0.69	0.65	0.63	0.981	0.817	532.0	<.001

#### 5.2.2 Correlation analysis

To gain an insight into which behavioral feature would characterize the best teachers–at least from the robot learning point of view–and whether this would influence the user experience we conducted a correlation analysis considering as behavioral variables the number of demonstrated skills and the number of answered questions, as teaching performance we considered the teaching score. Considering that questions are asked only regarding demonstrated appliances and effects and that the F2 score rewards more recall, we reasoned that users who demonstrated more tools as a strategy, rather than demonstrating the same tool with different food items or answering many questions regarding the same appliance, would achieve a higher score. We thus computed an “explorativeness” measure, considering the number of unique tools that were demonstrated. The pairwise correlation analysis among behavioral and subjective measures is presented in Table 3. As it can be noted, the highest positive correlation with the teaching score was with the explorativeness, suggesting that people who sampled more appliances for their demonstrations also achieved higher scores. The score also strongly correlated with the number of demonstrated skills and moderately with the number of answered questions. As said, this could be expected from the F2 formulation, yet this was not known to the users and recall is at the same time heavily penalized by missing generalizations (false negatives), which are reduced also by answering questions, as a viable strategy. Yet both score and the number of demonstrated skills appear most correlated with explorativeness and only loosely or not significantly with the number of questions. None of the behavioral measures was correlated to the subjective measures. Apart from some moderate correlation between UES subscales (PU and RW) with the general scale, the moderate correlation between the THRI and the SIPAS scales appears of interest, suggesting that the more users felt they understood the system, the more they trusted it.

**TABLE 3:** Correlation Table for the first interaction. After descriptive values for each variable, pairwise Spearman’s ρ are reported, along with the corresponding p -values, after Holm-Bonferroni correction. Significant correlations are highlighted in bold font.

	M	SD	Coefficient	1	2	3	4	5	6	7	8	9	10
1. skills_shown	3.30	1.70	ρ	—									
			p-value	—									
2. questions_asked	10.79	7.85	ρ	0.431	–								
			p-value	0.443	–								
3. score	0.21	0.10	ρ	**0.762**	**0.581**	–							
			p-value	<0.001	0.0016	–							
4. explorativeness	2.64	1.22	ρ0.001	**0.905**	0.467	**0.846**	–						
			p-value	<0.001	0.225	<0.001	–						
5. UES	3.58	0.36	ρ	−0.076	−0.033	−0.027	−0.04	–					
			p-value	1.0	1.0	1.0	1.0	–					
6. UES_FA	3.41	0.47	ρ	0.099	0.422	0.040	0.062	0.357	–				
			p-value	1.0	0.492	1.0	1.0	1.0	–				
7. UES_PU	3.43	0.67	ρ	−0.130	−0.366	−0.095	−0.054	**0.628**	−0.144	–			
			p-value	1.0	1.0	1.0	1.0	0.004	1.0	–			
8. UES_RW	3.88	0.68	ρ	−0.148	0.038	0.003	−0.059	**0.650**	0.025	0.123	–		
			p-value	1.0	1.0	1.0	1.0	0.002	1.0	1.0	–		
9. SIPAS	3.98	0.94	ρ	0.412	0.363	0.355	0.428	0.531	0.209	0. 318	0.242	–	
			p-value	0.572	1.0	1.0	0.454	0.057	1.0	1.0	1.0	–	
10. THRI	0.67	0.15	ρ	0.328	0.112	0.211	0.351	0.118	−0.143	0.252	0.078	**0.553**	–
			p-value	1.0	1.0	1.0	1.0	1.0	1.0	1.0	1.0	0.033	–

### 5.3 Second interaction

Considering the small number of participants who engaged with the system more than once, we here consider only whether significant differences could be assessed between the first and second interactions for the 16 returning users and test these with the Wilcoxon signed-ranked test.

#### 5.3.1 Subjective measures

Considering the data from users who interacted at two different time points (N = 16), differences in UES, SIPAS, and THRI scores between the first and the second interaction were all not significant (all p≫.05 ), but all three scores slightly declined, apart from the UES_RW subscale (UES: $M_{t2-t1}=-0.08,SD=0.67$ ; UES_FA: $M_{t2-t1}=-0.04,SD=0.80$ ; UES_PU: $M_{t2-t1}=-0.21,SD=0.88$ ; UES_RW: $M_{t2-t1}=0.02,SD=1.15$ ; SIPAS: $M_{t2-t1}=-0.34,SD=1.36$ ; THRI: $M_{t2-t1}=-0.07,SD=0.18$ ).

#### 5.3.2 Behavioral measures

Behavioral measures considered for the first interaction (the number of demonstrated skills, the number of asked questions, and the explorativeness) showed no significant change in the second interaction (all p≫.05 ). The score increased on average by 0.08 (SD = 0.05) w.r.t. the first interaction.

### 5.4 Free-form comments

After the questionnaires, participants could fill a final text box with free-form comments regarding their interaction with system. 17 comments were left, in some cases addressing more than one issue. Five comments were rather positive, either stating it was better than they expected or acknowledging that some practice was needed (*“No comment in particular. The system was a bit unintuitive for me at the beginning, but I guess there is always some learning curve “*). Most comments (7) were about the reliability of the AR action recognition, which at times made manipulations frustrating (*“It did not really work well for me. The visual representation of my hand kept disappearing, so I could not interact properly.“*; *“I was not able to teach anything because of VR troubles (opening, picking up things did not work consistently), had nothing to do with the robot.“*, said the only participant who managed to teach just one skill), hence representing a technical limitation of our system. A number of comments revealed a limited understandability of our system (*“Several times I taught something but the robot told me it did not learn anything”*) or explainability regarding why plan execution failed (*“it is not clear why some plan fail sometimes. Explanations would be nice to have”*). A couple of users mentioned that it was difficult to relate the standard questionnaires to their interaction with the system (e.g., *“Some questions are hard to answer because it is not just the robot’s performance that is in question, but rather our collaboration (e.g., when it comes to “have errors”, this also refers to how well I taught him.“*), referring to one item in the THRI questionnaire), or that even the questions posed by the robot were not straightforwardly answerable (*“I gave answers that might not be that helpful. For instance, a microwave can be used to heat an egg, but I would not recommend it. However, I was only asked if it could be used.“*).

## 6 Discussion

In this study, we proposed a robot training system integrating continual learning, task planning, and explainable HRI in AR. We then conducted an exploratory study to investigate how novel users invited to interact repeatedly perceived such a framework. The closed-world and limited ontology used in the scenario allowed users to teach and test the system in short episodes, nevertheless, it made possible to teach high-level semantic skills in the form of real-world tasks, rather than trajectories or objects to recognize. The framework purposely integrated an AR system, which allowed the user to manipulate objects in a virtual scene, and a robotic system that was able to learn from these demonstrations, interact with the user by asking questions to expand the learned skills, and, upon request, produce a semantically similar plan. This was achieved by seamlessly integrating different systems and avoiding Wizard-of-Oz solutions since we were interested in understanding how the user experience would be affected by the system’s actual performance. The stability and relative ease of use of the system allowed the experiment to run unsupervised most of the time, with users familiarizing themselves with the virtual scene.

The main results from questionnaire data show that the system is positively perceived across dimensions concerning engagement, trust, and understanding, with such perception staying stable across the first and second interactions. Hence, at least for the first two interactions the novelty did not wear off. Users who explored the scene more while teaching and engaged in question-answering were able to increase the robot’s knowledge more effectively. Moreover, while generally, the variance was low on the overall engagement scale–suggesting that the user experience was pretty consistent across participants in that regard, up to some outliers–there was a larger spectrum of experiences concerning understanding and trust. The moderate correlation between understanding and trust further substantiates the intuition that these two constructs often evolve together. Understanding implies the formation of a correct mental model that reduces the mismatch with expectations by increasing predictability and thus trust in the functioning of the learning system (Hoff and Bashir, 2015; Beierling et al., 2024). To sum up, while the system proved feasible (w.r.t. RQ1, RQ2, RQ3), both hypotheses (HP1 and HP2) were disproved or were not enough substantiated. The unsupervised nature of the study and the overall system limitations (as deduced from the free-form comments) led to a low return-rate, limiting insights into the evolution of the user experience and teaching dynamics. This is *per se* an insight which night be of interest to the community, since most studies prescribe specific interactions patterns under experimenter’s supervision: this helps with comparative analyses across participants and sessions but does not tell much about how users would spontaneously interact with learning systems. At the same time, this exploratory study suggests still that other aspects of the interaction need deeper consideration to scaffold the user’s understanding and willingness to engage with similar systems, which is what we discuss next as general lessons learned.

### 6.1 Functional vs hedonic aspects

While every participant was able to teach at least one skill and to answer related questions and they all considered the overall experience rewarding (according to the UES_RW scale, cf. Figure 8), still the returning rate was just half of the participants, limiting the insights on long-term teaching and learning. This can have a number of motivations, not all related to the system. This was presented as a voluntary user study in a research institute. The pool of invited participants is constituted by around 60 research staff and 10 administrative and tech support staff. While the turnout for the first interaction was relatively high, this might have been motivated by interest in coworkers’ research, curiosity to try out the AR interaction, and general professional interest in robots and technology. Still, people did not get compensated for their participation (other than with a treat), and after getting an idea of what the system was about and what it could do, they were perhaps not incentivized to further invest time in it. This can also hint at a trade-off between utilitarian and hedonic aspects of the proposed experience (De Graaf et al., 2016; Hu, 2021). While the overall framework was engaging, the robotic system was proposed in the context of a service robotics scenario, but the demonstration and plan executions happened all on a virtual level, bringing no real benefit to the user. Thus the hedonic aspects of playing in the AR and increasing the knowledge score, however engaging, were not motivating enough in the long term (De Graaf et al., 2016).

### 6.2 Beware of the cognitive workload

While the Hololens is relatively easy to wear and adjust, and the general interaction with the system had a relatively low learning curve, first-time users needed some introduction to the system and there were several things to keep in mind at any time, including taking into account the Hololens technical limitations and indications about how to operate the system, manipulate objects in the AR without haptic feedback and have the robot learn and plan effectively. Most frequent issues were revealed by pilot testing with project coworkers not familiar with the overall framework, thus, as described in Sec.4 information and tips were provided by means of multiple sources (a 6 min video tutorial, flashcards on the lobby wall, a command ‘cheat sheet’ in the scene). Open-form comments still revealed that not all participants were able to manipulate objects correctly or took into account the limited field of view of the Hololens display and camera. This means that the instructional materials were not always thoroughly followed or that the sheer amount of information might have contributed to increasing the extraneous cognitive workload (Hollender et al., 2010; Kosch et al., 2023), i.e., workload connected to the way instructional material is provided to the learners. In a previous study with a similar AR interface (Belardinelli et al., 2023), NASA-TLX scores had revealed a relatively low mental effort, still, in that case, the scene was simpler, and being in a controlled laboratory experiment provided optimal light conditions and the possibility for the experimenter to help promptly with technical issues. Here, while we provided as many reminders as possible, also inside the AR, the interface was not adaptive, would not detect the user struggling with some functionality, and would not provide support proactively, or suggest where to look for information, which is something future developments of the system should take into account.

### 6.3 Enhance technical explainability

While the robot made the tutor aware of what it had learned, and multiple graphical cues were given as to what it understood of the scene, no clear indication was given as to when and how the robot was generalizing knowledge. The only way to check whether the robot would be indeed able to come up with a plan for a demonstrated skill with a different food item was to ask for it. In principle, if the system got a demonstration related to some food item and a positive answer to a question about the same skill with another food item in the same WordNet category, it should be able to generalize and therefore, the closest items in that respect were asked first. Still, participants were not privy to such knowledge representation. Moreover, while the score increase gave a sense of progress, it was difficult for participants to estimate the gained knowledge. On the one hand, we avoided giving away too many technical details in order 1) to keep instructions short and clear, especially for users with no robotics background, and 2) to encourage users to explore more and figure out for themselves what worked best. On the other hand, the system could have given hints about the abstracted knowledge or suggested promising directions (e.g., “I’ve learned that the toaster makes bread hot and crispy, if you answer two more questions or give more demonstration I’ll learn that baked goods can be used with the toaster”) or, after a plan for a request was not found or had failed for low-level planning reasons, it could have asked for more input (e.g., “Can you demonstrate this specific task? I have a problem with opening the microwave”). This would constitute a form of teaching guidance (Cakmak and Thomaz, 2014) applied to interactive robot learning. In this way, even teachers with no clear mental model of the robot’s learning would have been pointed in the right direction. While providing more technical insights might be in some cases at odds with the previous guideline on reducing mental workload, timely sharing of technical information as a balance of instructions and online (multimodal) feedback could prove extremely beneficial in complex learning systems, as proposed by Hindemith et al. (2021) and Habibian et al. (2023). In future work, an optimal trade-off between general instructions and online visual feedback about internal learning states should thus be investigated.

In all these points, exciting progress is being boosted by approaches making use of Large Language Models (e.g., Joublin et al., 2024; Tanneberg et al., 2024; Shi et al., 2024), but integration with grounded perception, action, and user understanding will be the next challenge. The lessons learned here should be taken into consideration in such contexts.

## 7 Conclusion

While future robots will come with numerous pre-trained skills, teaching specific tasks will help users expand the robot’s repertoire and personalize robots for their own needs and preferences. This will need to happen easily and intuitively for long-term interaction. Here, we contributed an end-to-end framework to train robots in AR where common ground can be more easily shared between tutor and trainee, and the robot’s ability to use the gained knowledge for similar tasks can be straightforwardly tested. We presented an exploratory study that allowed participants to test the framework independently. The system proved to be overall engaging and provided enough explainability to leave teachers with a sense of understanding and–to some extent–trust. However, such an experimental framework also highlighted the still very current challenges both in designing repeated interaction studies that take place in the wild rely on live and spontaneous participation and in devising interactions that effectively balance functional and hedonic aspects while communicating the robot’s internal states in an informative, actionable way. In conclusion, our study indicates that interactive task learning should rely on an integrated architecture that makes transparent to users the way the system learns and guide them in the whole process, minimizing the cognitive load from interaction interfaces and teaching feedback.

## Data Availability

The raw data supporting the conclusions of this article will be made available by the authors, without undue reservation.

## Footnotes

1 In the present study, all physical objects and the robot itself are presented as holograms for logistic simplicity, still, they all are virtual replicas of real counterparts that can be used as anchors for AR visualizations. Even so, AR still allows to perceive the real, familiar environment behind the holograms, alleviating possible discomfort from permanence in VR (Vovk et al., 2018) and adding to the illusion of realism.

2 We assume the robot relies on hypothetical sensors to detect such changes. In practice, such events are simulated in the virtual scene by changing related elementary attributes of the food when turning on an appliance or if the tea bag is put in a liquid, respectively.
